# Surgical revision for stump problems after traumatic above-ankle amputations of the lower extremity

**DOI:** 10.1186/s12891-015-0508-3

**Published:** 2015-03-05

**Authors:** Kemin Liu, Tao Tang, Anqin Wang, Shouchang Cui

**Affiliations:** School of Rehabilitation Medicine, Capital Medical University, Beijing, China; Department of Orthopedics and Rehabilitation, Beijing Charity Hospital, No.10 Jiaomenbei Road, Fengtai District 100068 Beijing, China

**Keywords:** Lower extremity, Amputation, Traumatic, Stump problems, Surgical revision

## Abstract

**Background:**

Stump problems (SPs) secondary to traumatic lower limb amputation had a crucial influence on amputees’ ability to return to living and work. The purpose of this study was to investigate the surgical management strategies of the SPs after above-ankle amputation of the lower limb secondary to trauma.

**Method:**

A cohort of clinical cases, who were troubled by SPs after above-ankle amputation following trauma, had undergone revision surgery of the stump and was analyzed retrospectively. Various factors were noted like sex, unilateral or bilateral, amputation type, and causes of trauma. Different SPs like excess soft tissue (where a considerable amount of soft tissue interposed between the rigid elements which hindered the fitting of a prosthesis), scar, ulcers, neuromas, and bone spurs were taken as dependent variables. The relationship between factors and SPs was analyzed.

**Results:**

A total of 80 stumps were treated surgically. The frequency of excess soft tissue in above-knee amputation cases was higher than that in below-knee amputation (p = 0.007). Bone spur occurred more frequently in the unilateral amputation than in bilateral ones (p = 0.018). There was a significant difference in the ADL scores between admission and discharge (p = 0.000).

**Conclusion:**

Stump problems secondary to traumatic lower limb amputation had crucial influence on amputees’ ability to return to living and work, appropriate evaluation and timely surgical revision showed excellent results.

## Background

Stump problems (SPs) are very common complications in patients, who have suffered from traumatic amputation, and often impede prosthetic fitting and weight-bearing [1]. SPs include stump skin scar or ulcer, delayed wound healing, excess soft tissues, prominent bone under skin, and stump pain caused by spurs or neuroma. The aims of this retrospective study were to examine factors that influence the occurrence of SPs and to discuss the management strategies of surgical revisions.

## Methods

### Study design and population

A retrospective cohort design was used. The study population consisted of 248 amputees (including upper and lower limbs) who were admitted to a rehabilitation program from November 1992 to August 2008. Among these patients, 72 received appropriate surgical operations on 80 residual limbs due to SPs after an above-ankle amputation secondary to trauma. Ethics approval was obtained before initiation of the study from the Ethic Committee of Beijing Bo Ai Hospital. Consent from patients was obtained for their images to be published.

The following variables were extracted from the medical records: age, gender, mean duration of hospitalization (days), amputation types, months from primary amputation to stump revision, causes of trauma for amputation, soft tissue coverage (excess soft tissue, obvious scar, ulcer, neuroma), bone protrusion and spur, and unilateral or bilateral amputation. The Activities of Daily Living (ADL) score was also measured using the Barthel index (BI) [2] by trained physicians at admission and discharge. The ADL scores ranged between 0 and 100.

### Surgical procedures

**Figure 1:**
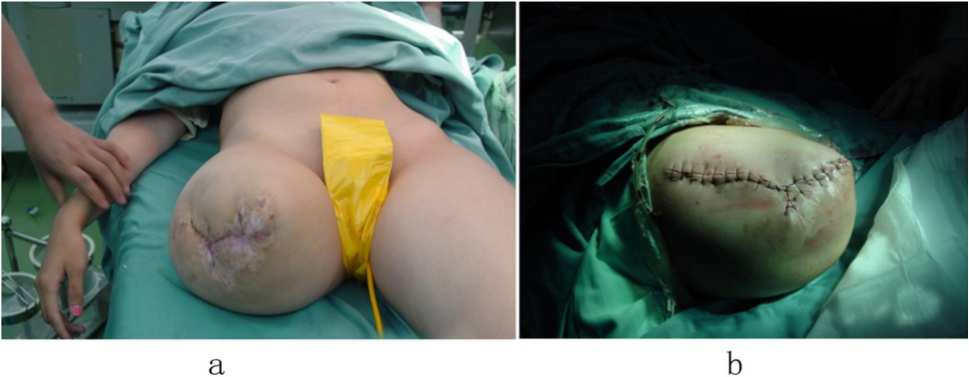
**Excess soft tissue and scars of the stump affected prosthetic wearing and weight-bearing (a); the shape of residual limb was improved significantly after stump revision (b).**

**Figure 2:**
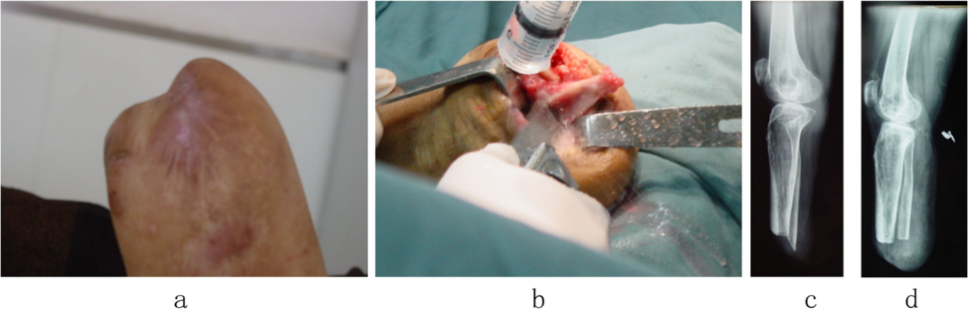
**Scar, longer fibula, stump pain influence weight-bearing.** Bone protrusion and scar on weight-bearing impeded the fitting of prosthesis (**a)**; dissection of the longer fibula and scar,trim the bone end smoothly and then suture with myoplasty **(b)**; a sharp fibular end showed on the X-ray before operation **(c)**; the fibula residual end was smooth and soft tissue was cylindrical on the X-ray after operation **(d)**.

**Figure 3:**
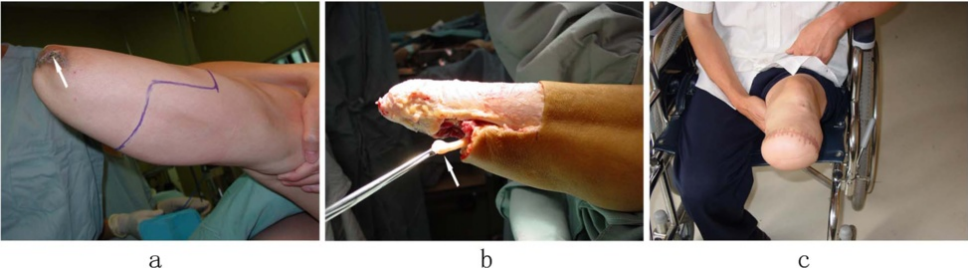
**A longer but less ideal residual limb after the amputation of the leg, with conical shape and repeated ulceration stump (arrow) (a); the standard leg amputation procedures were performed with resection of the neuroma (arrow) at the same time (b); the stump was cylindrical with good soft tissue coverage (c).**

The surgical procedures were performed under continuous epidural anesthesia with tourniquets. Plastic surgery was done when the soft tissue coverage was poor, including for excess soft tissue (Figure 1) or obvious scars and ulcers (Figures 2a and 3a). If the defect in the soft tissue was assessed to be too large to be covered by surrounding healthy skin after excision, then the patients or his family members were asked to assist with the manipulation procedures of pushing and pulling the scar and surrounding healthy skin, or water balloon dilators were implanted under the healthy skin surrounding the scar to dilate the skin. Surgery was not carried out until the laxity of the surrounding healthy skin was identified to be adequate to cover the defect. Stamp skin grafting was avoided because of its poor wear resistance, which might cause skin rupture when the prosthesis was installed. If stump pain was determined to be caused by the bone spur or neuroma, surgical resection (Figures 2b and 3b) was then performed. The surgical resection of a neuroma was achieved by sharp dissection and nerve end ligation, followed by embedding the nerve end with epineurium. The stump bone spur was removed under direct vision, and the bone end was rasped to a round, smooth contour (Figure 2b). If the stump was identified to be conical, either myodesis or myoplasty was performed, and the bone was adequately shortened to create cylindrical or cylindrical-like residual limbs (Figure 3c).

**Figure 4:**
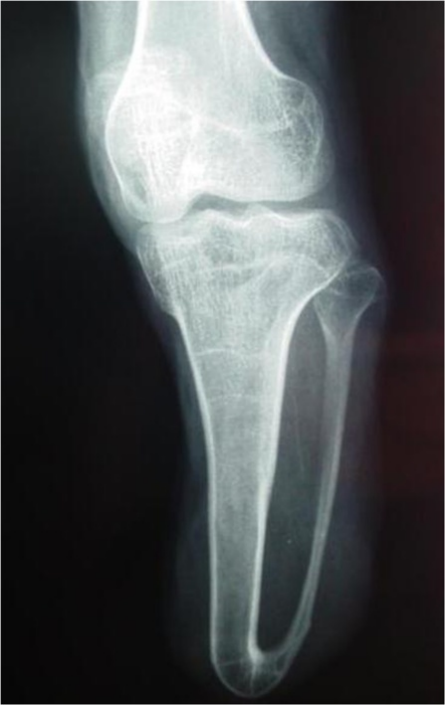
**The synostosis of tibia and fibula was achieved using Ertl’s technique with the formation of a bone bridge.**

If there was a longer and prominent fibula in the residual lower leg, an osteoplastic procedure was performed, that has been well documented in previous reports [3-7]. After the fibular shortening, the opposite surfaces of the fibula and tibia were coarsened to form a scaly bed using a bone chisel; an iliac bone block was grafted between them; and a screw was then used to fix them for synostosis. Alternatively, Ertl’s osteoplastic procedure [6] was also performed to obtain a bone bridge fusion (Figure 4) by making a flap of periosteum, to which chips of bone remained attached.

If there was ostemyelitis or a deep infection in the residual limb, complete debridement and continuous irrigation drainage suction was performed until the infection was resolved. In order to facilitate absorption of the stump swelling and to prevent flexion contracture, elastic bandages or plaster were routinely used [4]. If SPs could not be solved by any of the surgical procedures mentioned above, a revision amputation would then be performed proximally (Figure 3).

### Statistical analysis

Data analysis was performed using the SPSS program for Windows, version 11.5. Continuous measures were described as mean ± SD and compared using the Student’s *t*-test or one-way analysis. Categorical variables were described by percentages and compared using the Chi Square test. The significant level was set as 0.05. Gender, unilateral or bilateral amputation, amputation level, causes of trauma were taken as factors. The relationship between factors and stump problems was analyzed (Tables 1, 2, 3 and 4).

**Table 1:** **The relationship between gender and stump problems**

**Gender**	**Cases**	**Time** ^**a**^	**Excess soft tissue**	**Scar**	**Ulcer**	**Neuroma**	**Spur**
			**N(%)**	**N(%)**	**N(%)**	**N(%)**	**N(%)**
Male	47	1.10 ± 0.56	24(30.0)	42(52.5)	14(17.5)	38(47.5)	56(70.0)
Female	25	1.06 ± 0.68	8(7.8)	17(13.65)	7(4.55)	13(12.35)	17(18.2)
Statistics		0.455	0.011	2.564	2.369	0.097	0.391
P value		0.650	0.917	0.109	0.124	0.750	0.532

^a^Mean months from primary amputation to stump revision, logarithmic transformation was performed to obtain normal distribution.

**Table 2:** **The relationship between amputation side and stump problems**

**Amputation side**	**Cases**	**Time** ^**a**^	**Excess soft tissue**	**Scar**	**Ulcer**	**Neuroma**	**Spur**
			**N(%)**	**N(%)**	**N(%)**	**N(%)**	**N(%)**
Unilateral	59	1.11 ± 0.58	16(16.5)	31(28.88)	10(9.65)	28(26.13)	34(38.5)
Bilateral	13	1.09 ± 0.49	8(7.5)	11(13.12)	4(4.35)	10(11.87)	22(17.5)
Statistics		0.137	0.069	1.054	0.057	0. 820	5.610
P value		0.892	0.792	0.305	0.812	0.365	**0.018**

^a^Mean months from primary amputation to stump revision, logarithmic transformation was performed to obtain normal distribution. Bold numbers mean P <0.05.

**Table 3:** **The relationship between amputation type and stump problems**

**Amputation type**	**Cases**	**Time** ^**a**^	**Excess soft tissue**	**Scar**	**Ulcer**	**Neuroma**	**Spur**
			**N(%)**	**N(%)**	**N(%)**	**N(%)**	**N(%)**
Thigh	32	0.94 ± 0.46	15(9.6)	14(16.8)	6(5.6)	18(15.2)	20(22.4)
Leg	48	1.12 ± 0.59	9(14.4)	28(25.2)	8(8.4)	20(22.8)	36(33.6)
Statistics		2.21	0.792	0.305	0.812	0.365	0.018
P value		**0.03**	**0.007**	0.201	0.810	0.201	0.232

^a^Mean months from primary amputation to stump revision, logarithmic transformation was performed to obtain normal distribution. Bold numbers mean P <0.05.

**Table 4:** **The relationship between trauma causes and stump problems**

**Trauma causes**	**Cases**	**Time** ^**a**^	**Excess soft tissue**	**Scar**	**Ulcer**	**Neuroma**	**Spur**
			**N(%)**	**N(%)**	**N(%)**	**N(%)**	**N(%)**
Traffic accident	45	1.17 ± 0.65	15(13.5)	23(23.63)	7(7.88)	24(21.38)	32(31.5)
Machine winding	21	1.04 ± 0.38	2(0.35)	7(7.35)	2(2.45)	5(6.65)	9(9.8)
Crash injury	14	0.99 ± 0.41	7(6.3)	12(11.03)	5(3.68)	9(9.98)	15(14.7)
Statistics		0.841	1.995	0.251	0.797	1.575	0.265
P value		0.435	0.369	0.882	0.671	0.455	0.871

^a^Mean months from primary amputation to stump revision, logarithmic transformation was performed to obtain normal distribution.

## Results

The study population consisted of 72 amputees with 80 residual limbs and 47 men and 25 women aged between 9 and 20 years (mean age 28.8 ± 12.4). Of the 80 residual limbs, 32 were above-knee amputations (5 knee disarticulation and 2 hip disarticulation were included in the above-knee amputation group because of difficulty for analysis as a single group), and 48 were below-knee amputations. With respect to causes of trauma, 45 (56.25%) patients were traffic accidents, 21 (26.25%) patients were crash injuries (mine collapse etc.), and 14 (17.5%) patients were machine winding accidents. The criteria of clinical evaluation and analysis of the stump problems are shown in Table 5. The mean duration from primary amputation to surgical revision of stump problems was 32.7 months.

**Table 5:** **Evaluation criteria of stump problems**

**Category**	**Content**
**Anatomy**	Skin: scar, folds, folliculitis, ulcer, fistulas etc.
	Muscle: loose, without myodesis and myoplasty
	Nerve: neuroma, adhesion of nerve
	Bone end:spur, abduction or mecism of fibula
**Pain nature**	Stump pain (4 types [4]): nerve stimulation, circulatory disturbance, muscle anomalous tension, bone spur
	phantom limb pain:complicated reasons, not fully clear
**Shape of stump**	Conical
	Cylindrical
	Irregular
**Soft tissue coverage**	Extensive skin grafting,including stamp skin grafting
	Bone protrusion subcutaneously with muscle retraction
	Excess soft tissue
	Heavy scar
**Primary amputation**	Emergent operation for amputation
	Failure of limb salvage
	Normal healing after amputation
	Necrosis, infection, delayed healing
**Infection**	Yes, companied with ulcer, fistulas, osteomyelitis etc.
	No

Among the 80 stump revisions in this study, 55 patients (66.3%) did not undergo myodesis in the primary amputation; 42 (52.5%) patients had heavy scar; 38 (47.5%) patients had a neuroma; 24 (30%) patients had excess soft tissues; and 14 (17.5%) patients had ulcers. For surgical revision strategies, 14 patients underwent tibiofibular synostosis (8 Ertl’s technique, 6 bone grafting and screw fixation), and 12 patients had a revision more than twice because of neuroma, bone spur, or heavy scar. Thirteen patients (21 limbs) were bilateral amputees. The standard leg amputation procedures were performed for five cases. No cases required advancement of their amputation level from below knee to above knee. The average length of hospital time for patients with or without medical insurance was 220.2 and 103.6 days, respectively. Statistical analysis results are shown in Tables 1, 2, 3 and 4.

ADL scores were collected on forty-one patients both at admission and discharge. There was a significant increase in ADL scores at discharge (95.40 ± 3.92) compared to that at admission (85.31 ± 7.24) (t = −11.536, P <0.001).

## Discussion

The main causes of amputation in this study were high-energy injuries with 56.25% caused by traffic accidents, 26.25% by crash, and 17.50% by machine winding injuries. The patients in this study were mostly young adults with a median age of 28.8 ± 12.4, mainly in their most active stages of life; the importance of obtaining functional amputation stumps cannot be overemphasized. Studies demonstrated that stump problems after lower limb amputations are important factors to impede proper prosthetic fit and the functional outcome of amputees [1,8-12]. The average duration of hospitalization for patients with medical insurance (220.2 days) was longer than those without insurance (103.6 days).

With regards to the functional amputation stumps, Burgess et al. [13] put forward the concept of functional residual limb (i.e. the stump should play a role as locomotive and sensory organ like human’s feet), while the artificial limb functions like shoes. Based on this concept, Cui et al. [4] further proposed that residual limbs should be classified as ideal and less-ideal stumps. Both concepts were proposed to emphasize and highlight that amputation should be regarded as a functional reconstruction procedure rather than just simple limb dissection after the failure of limb salvage treatment. According to the randomized study by Wadwhani [14], 43% of lower limb amputees had stump problems. Among the 248 amputees who were admitted for rehabilitation in this study, 72 (29.0%) patients who had above-ankle amputations secondary to trauma received stump revision. Stump problems are very common in lower limb amputees. The principal aim of the present study was to examine the relationship between the factors of gender, unilateral or bilateral amputation, amputation level, causes of trauma, and stump problems.

### I. The evaluation of stump problems

The causes of stump problems are complex and multi-factorial, such as anatomical structures, nature of pain, stump shape, soft tissue coverage, the conditions of primary amputation, and infection (Table 5). A comprehensive consideration of all these factors will greatly aid accurate judgment, evaluation, and appropriate treatment of SPs.

**Figure 5:**
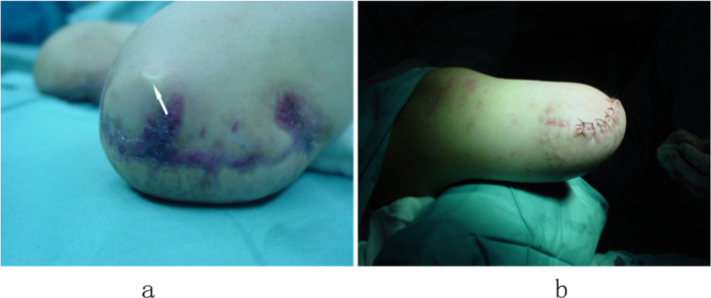
**Leg amputation without myodesis and myoplasty, tibial end protruding under the skin (arrow), heavy scar, weight bearing pain (a); Surgical revision included osteoplasty, myodesis and scar excision was performed, a satisfactory weight-bearing surface is achieved (b).**

In this study, issues related to soft tissue are the main cause of the stump problems (Tables 1, 2, 3 and 4), which is consistent with the report by Rotter et al. [15]. Fifty-three (66.3%) amputation limbs did not undergo myodesis in primary amputation (Figure 5a); 42 (52.5%) had excessive scar; 38 (47.5%) had a neuroma; 24 (30%) had excess soft tissues; and 14 (17.5%) had skin ulcers. Twelve stumps required revision surgery more than twice, primarily from stump pain from neuromas and bone spurs. Five patients, who felt discomfort, recurrent ulceration, or scab and callus formation after wearing their prosthesis, received re-amputation surgery for their residual limbs. Of the five cases, two had been amputated in the distal third portion of the lower leg that led to a longer stump but poor soft tissue coverage (Figure 3a); three suffered from tissue necrosis and infection due to incomplete debridement or stamp skin grafting after severe initial injuries. The standard lower leg amputation procedures were performed, and a functional stump was obtained (Figure 3b & c). Compared with the longer but poor wear-resisting leg stumps, an idea leg stumps should be pursued at the stage of primary amputation. The well wear-resisting residual limbs developed three months post-revision. The patients could wear the prosthesis with successful fitting, the walking pattern was improved obviously.

The data in the present study also suggested that stump problems were usually related to iatrogenic factors: a lack of understanding of the theoretical knowledge and the surgical techniques about amputation and rehabilitation. A large proportion of cases (66.3%) in this group did not get a myodesis or myoplasty at the primary amputation. Considering the cost and suffering to keep longer but less ideal residual limbs for the patients, the importance of achieving a satisfactory weight-bearing stump, even if a little shorter but with enough and healthy soft tissue coverage, cannot be overemphasized (Figure 3c). The management of skin, muscles, nerves, blood vessels, or bone should follow modern amputation ideas and technology [3,16].

### II. The influence factors on the stump problems

The relationship between six common stump problems and factors such as gender, unilateral or bilateral amputation, amputation level and causes of trauma were analyzed. We found that the stump problems demonstrated no significant relationship with gender and trauma causes. The mean time from primary amputation to stump revision for the below-knee amputation was significantly longer than that of the above-knee amputation (p = 0.03), probably because a lower leg prosthesis has better compensatory function and relatively lower risk to injure the stump than a thigh prosthesis. The frequency of excess soft tissue in a thigh stump was significantly higher than that of a lower leg stump, and the difference was statistically significant (p = 0.007). The presence of a considerable amount of soft tissue interposed between the rigid elements and the absence of ligamentous or muscle prevented the successful fitting of the prosthesis (Figure 1a). Fifty-six residual limbs (70%) received revision surgery for bone spurs, which were observed more frequently in unilateral amputation than in bilateral amputation with significant statistical difference (p = 0.018). The potential reason may be that the patients with unilateral amputations use their stumps more frequently.

BI scores were obtained for 41 patients at both admission and discharge, and the values at discharge (95.40 ± 3.92) were significantly higher than that at admission (85.31 ± 7.24) (t = 11.536, p < 0.001), indicating that stump revision and the related rehabilitation therapy improved amputees’ limb function. In this study, only the ADL scores at the time points of admission and discharge were collected and compared. Therefore, we could not analyze how long it took patients to improve their ADL scores. This is really a limitation and should be remedied in our future research.

### III. The surgical management of stump problems

Stump trimmed surgery, also called as stump revision [15], is currently a good solution to stump problems, for which prosthesis and rehabilitation programs are usually less effective. Our study suggests that grasping the key points of stump revision surgery and an optimized surgical strategy are of paramount importance to the outcomes of stump revision. Revision should not be carried out when there is too much scar tissue and too little healthy normal skin, until the surrounding healthy skin could fully cover the wound through the techniques of pulling skin or water balloon dilation. Free skin grafting and free skin flap is not the first choice. There were six stumps with heavy scars in this study, water balloon dilation technique promoted coverage of the wound with adequate skin after the resection of the scar. After the amputation of the lower limb, longer or abduction fibula deformities will seriously affect the prosthesis matching and weight bearing. Fibular shortening and tibia-fibula synostosis are standard treatments. The synostosis could be achieved through Ertl’s procedure [6] or lag screw technique with or without bone grafting between tibia and fibula. Fourteen patients in this study have gained satisfactory tibia-fibula synostosis by using one of these two methods (Figure 4). For neuromas, we recommend using sharp dissection and nerve end ligation, followed by embedding the nerve end with epineurium. By not performing a myodesis or myoplasty during the primary amputation, conical stumps and bone protrusions under the skin can result (Figure 5a). Standard myodesis and myoplasty procedures were performed in stump revision (Figure 5b).

In short, stump problems after traumatic above-ankle amputations are significantly influences the functional rehabilitation of the residual stump. *Encouraging outcomes are achieved through appropriate surgical revision management in this series*. In China, the related science and clinical research in this area is still insufficient [16], further study is warranted.

## Conclusions

***In this series***, stump problems secondary to traumatic lower limb amputation produced an effect on amputees’ ability to return to living and work, appropriate evaluation and timely surgical revision showed excellent results.
